# The remote exercise monitoring trial for exercise-based cardiac rehabilitation (REMOTE-CR): a randomised controlled trial protocol

**DOI:** 10.1186/1471-2458-14-1236

**Published:** 2014-11-28

**Authors:** Ralph Maddison, Jonathan C Rawstorn, Anna Rolleston, Robyn Whittaker, Ralph Stewart, Jocelyne Benatar, Ian Warren, Yannan Jiang, Nicholas Gant

**Affiliations:** National Institute for Health Innovation, University of Auckland, Private Bag 92019, Auckland, 1142 New Zealand; Department of Sport and Exercise Science, University of Auckland, Auckland, New Zealand; The Cardiac Clinic, Tauranga, Bay of Plenty, New Zealand; Department of Cardiology, Auckland City Hospital, Auckland, New Zealand; Department of Computer Science, University of Auckland, Auckland, New Zealand

**Keywords:** mHealth, Telemonitoring, Remote sensing technology, Exercise training, Peak oxygen uptake, Coronary heart disease, Smartphone, App

## Abstract

**Background:**

Exercise is an essential component of contemporary cardiac rehabilitation programs for the secondary prevention of coronary heart disease. Despite the benefits associated with regular exercise, adherence with supervised exercise-based cardiac rehabilitation remains low. Increasingly powerful mobile technologies, such as smartphones and wireless physiological sensors, may extend the capability of exercise-based cardiac rehabilitation by enabling real-time exercise monitoring for those with coronary heart disease. This study compares the effectiveness of technology-assisted, home-based, remote monitored exercise-based cardiac rehabilitation (REMOTE) to standard supervised exercise-based cardiac rehabilitation in New Zealand adults with a diagnosis of coronary heart disease.

**Methods/Design:**

A two-arm, parallel, non-inferiority, randomised controlled trial will be conducted at two sites in New Zealand. One hundred and sixty two participants will be randomised at a 1:1 ratio to receive a 12-week program of technology-assisted, home-based, remote monitored exercise-based cardiac rehabilitation (intervention), or an 8-12 program of standard supervised exercise-based cardiac rehabilitation (control).

The primary outcome is post-treatment maximal oxygen uptake (V̇O_2_max). Secondary outcomes include cardiovascular risk factors (blood lipid and glucose concentrations, blood pressure, anthropometry), self-efficacy, intentions and motivation to be active, objectively measured physical activity, self-reported leisure time exercise and health-related quality of life. Cost information will also be collected to compare the two modes of delivery. All outcomes are assessed at baseline, post-treatment, and 6 months, except for V̇O_2_max, blood lipid and glucose concentrations, which are assessed at baseline and post-treatment only.

**Discussion:**

This novel study will compare the effectiveness of technology-supported exercise-based cardiac rehabilitation to a traditional supervised approach. If the REMOTE program proves to be as effective as traditional cardiac rehabilitation, it has potential to augment current practice by increasing access for those who cannot utilise existing services.

**Trial registration:**

Australian New Zealand Clinical Trials Registry

Study ID number: ACTRN12614000843651. Registered 7 August 2014

## Background

Cardiovascular diseases (CVD) are the leading cause of death worldwide - 30% (16.7 million) of total deaths globally [1]. The largest proportion of CVD mortality is attributed to coronary heart disease (CHD) [2, 3], the prevalence of which is projected to increase 16.6% by 2030 [4]. Thus there is an increasing need for secondary prevention strategies to reduce the impact of CHD. Cardiac rehabilitation (CR) is a complex secondary prevention intervention that aims to optimise cardiovascular disease risk reduction, promote the adoption and adherence of healthy behaviours and reduce disability among those with established CHD [5]. Secondary prevention guidelines recommend a multifaceted CHD risk management approach [6–8]; however exercise is consistently identified as an integral component of CR [5, 9–11], and its potential as a risk modification strategy extends beyond the effects of physical inactivity alone.

Numerous beneficial cardiovascular and metabolic adaptations enable exercise to concurrently target several established CHD risk factors including blood pressure, blood lipid profile, glucose metabolism, weight status and body composition [12]. Moreover, a Cochrane review [13] reported 13% (RR = 0.87, 95% CI 0.75, 0.99) and 26% (RR = 0.74, 95% CI 0.63, 0.87) reductions in all-cause and cardiovascular mortality, respectively, following exercise-only CR and comprehensive CR incorporating an exercise component. These findings are supported by several other meta-analyses [14–16].

Despite its documented benefits CR adoption is low in many countries [9, 17, 18], including New Zealand [19, 20]. Exercise adherence is also poor, with up to 36% attrition from supervised programs [21–24]. Factors commonly associated with sub-optimal participation include ill health, domestic responsibilities, and difficulty accessing supervised programs [25, 26]. Low accessibility is of particular concern as it is associated with higher levels of cardiac morbidity and mortality [27]. A recent HEART journal editorial [28] concluded that CR should not only focus on CHD risk factor modification and medication adherence but should also offer a range of different delivery options for people according to their preferences and needs to address the low levels of participation. One such approach that has been investigated for risk factor modification has been the use of telehealth, which involves telephone, internet, and videoconference communication between patient and health-care provider.

A systematic review of 11 telehealth trials (n = 3145) showed significant improvements on CHD risk factors including exercise adherence and volume, total cholesterol, high density lipoprotein cholesterol and systolic blood pressure [29]. While these findings support telehealth, research has been limited to land-based telephone, internet and videoconferencing technologies that confine participants to fixed locations. As such there is a need to explore technologies that support increased program flexibility. Mobile technologies, including the internet are therefore gaining increased research attention as an alternative approach to support behaviour change, clinical improvement, and improved social functioning [30].

Emerging evidence to date for mobile interventions for delivering healthcare and improving disease self-management (mHealth) is promising [30]. A number of systematic reviews support the delivery of mobile phone text messaging interventions [31–33] for achieving behaviour change across a range of behaviours and chronic conditions such as diabetes and asthma.

In terms of CHD, the recently completed HEART randomised controlled trial (n = 171) demonstrated a mobile phone text messaging and internet intervention was effective and cost-effective for increasing leisure time physical activity and walking, but was not effective for increasing maximal oxygen uptake over and above usual care in people with CVD at 6 months [34]. Compared to the usual care control group, the HEART intervention also significantly increased participants’ health-related quality of life (physical health domain), self-efficacy and motivation to be physically active. Structured exit interviews conducted with those who were randomised to the intervention showed the HEART intervention was well received, had positive effects on participant’s physical activity levels, and was not considered burdensome. Most (93%) participants read all or most of their text messages [35]. Whilst this trial demonstrated the feasibility and effectiveness of a text messaging intervention to increase physical activity levels, improvement is needed to ensure interventions achieve positive impacts on exercise capacity and other CVD risk factors. Such improvements include closer exercise monitoring to ensure participants meet the required frequency, intensity and duration to realise beneficial physiological adaptations.

Increasingly powerful mobile technologies, such as smartphones and wireless physiological sensors, may extend the capability of mHealth exercise-based CR (exCR) by enabling real-time remote exercise monitoring for those with CVD. The feasibility of remotely monitored exCR was recently demonstrated using a smartphone, ECG sensor and GPS receiver [36]. A six-week remote exCR program improved walking performance (comparable to traditional exCR), cardiac depression and physical health-related quality of life. Furthermore, system usability and reliability were rated highly by participants. These encouraging results demonstrate the feasibility of remote exCR; however, a randomised controlled trial is required to determine the benefits and harms associated with this approach.

### Aim

To compare the effectiveness of technology-assisted, home-based, remotely monitored exCR (REMOTE) to standard supervised exCR in New Zealand adults with a diagnosis of CHD.

### Hypotheses

The primary hypothesis is that the REMOTE program will be as effective at increasing exercise capacity compared to standard exCR. Secondary hypotheses are; the REMOTE program will result in similar improvements in other cardiovascular risk factors (blood lipid and glucose concentration, blood pressure, anthropometry) compared to standard exCR. The REMOTE program will result in greater exercise adherence compared to standard exCR.

## Methods/Design

The study design is a single-blinded, two-arm, parallel, randomised controlled non-inferiority trial. Given the established effectiveness of supervised exCR [12] the REMOTE program is unlikely to result in a substantially greater improvement in exercise capacity; however, it’s advantages in terms of greater reach and participant adherence highlight the appropriateness of a non-inferiority trial design. The protocol is in accord with the SPIRIT 2013 statement [37, 38], and the intervention is described according to the CONSORT-EHEALTH checklist [39].

### Eligibility and recruitment

Eligible participants are adults aged 18 years or more, with a diagnosis of CHD (angina, myocardial infarction, percutaneous coronary intervention or coronary revascularisation) within the previous six months. Participants are current outpatients who have been clinically stable for at least six weeks, are able to perform exercise, and can understand and write English. A Motorola (Moto G) smart phone is available on loan to participants in the REMOTE group. Participants who have been admitted to hospital with heart disease within the previous six weeks, have terminal cancer, are contraindicated for maximal exercise testing, have significant exercise limitations other than CHD, currently meet the recommendations for regular physical activity (150 min/week moderate to vigorous) [40], are currently participating in a supervised exercise program (including exCR), have a pacemaker or implantable cardioverter-defibrillator, or have contraindications for maximal exercise testing are excluded.

Eligible participants are identified by research nurses or research assistants from a large metropolitan hospital in Auckland, New Zealand (population 1.6 million) prior to discharge, through outpatient clinics and existing databases, as well as existing community CR education sessions. Those agreeing to participate are screened for eligibility and provided a study pack, which includes a participant information sheet and consent form. Contact details of interested participants are sent to the research team. Participants identified in hospital and through outpatient clinics are telephoned approximately one month after discharge or one week after initial contact, respectively, to confirm their interest in the study and schedule a baseline assessment. Eligible participants are also recruited by a research staff at an existing CR clinic in the Bay of Plenty, New Zealand. These participants have been discharged from hospital, are eligible to participate in CR but have not yet enrolled in a program. Interested participants are given a study pack and a baseline assessment is scheduled.

### Sample size calculation

The target sample size of 162 participants (81 per group) will provide 80% power at 2.5% level of significance (one-sided) to show that the REMOTE and standard exCR programs do not differ by more than ml⋅kg^-1^⋅min^-1^ on peak oxygen uptake (V̇O_2_max). This sample size is based on the assumption the standard exCR program will result in an increase of 2.4 ml⋅kg^-1^⋅min^-1^ (SD =2.7) in V̇O_2_max, and has been inflated to allow for 10% loss to follow up [12]. The non-inferiority margin was chosen because it is clinically significant and is associated with lower CV-mortality [41, 42].

### Ethics approval

Ethical approval for the trial was received from the University of Auckland Human Participants Ethics Committee (011021). Approval was also obtained from the Metropolitan Hospital’s Research Review Board.

### Randomisation and blinding

After written consent is obtained, baseline assessment is completed and then participants are randomised using sealed sequential, opaque envelopes. Participants are randomly allocated to either the control (traditional exCR) or intervention (REMOTE) arms at a 1:1 ratio, stratified by study site and sex. The allocation sequence is overseen by the project statistician (YJ). Assessors of the primary outcomes are blind to treatment allocation; however participants are not blind.

### Intervention

The REMOTE intervention is delivered over 12 weeks and comprises a personalised exercise prescription, real-time remote exercise monitoring, and behavioural support to increase exercise adherence (goal setting, exercise scheduling, overcoming barriers), delivered via smartphone. The intervention aims to have individuals participate in moderate to vigorous aerobic-based exercise for at least 30 minutes (preferably more), most days (≥5) of the week, in line with current recommendations [40] and American College of Sports Medicine (ACSM) guidelines [43]. Specific details are provided below.

#### Exercise prescription

An individualised exercise prescription, based on personal preferences and current exercise capacity, is a core component of the intervention. Following ACSM guidelines for exercise in cardiac patients [43] participants are provided with a weekly prescription detailing exercise duration, frequency and intensity, via a smartphone application (app) designed for this study. The prescribed exercise intensity is sufficient to induce a “training effect”, yet below a metabolic load that evokes abnormal clinical signs or symptoms. Exercise duration is increased according to symptoms and clinical status. Exercise intensity is increased gradually as tolerated [44]. Progression of exercise prescription components occurs in the following order: duration, frequency, then intensity [44]. Participants are taught and encouraged to use ratings of perceived exertion (RPE) and the heart rate reserve method (HRR) to achieve the desired intensity. During early stages the level of intensity targets an RPE of 11 to 13 (“fairly light” and “somewhat hard”; 6–20 scale) [45] and/or 40% to 50% HRR. During the latter stages the level of intensity targets an RPE of 13 to 15 (“somewhat hard” to “hard”) and/or 55% to 65% HRR. The preferred mode of exercise is walking, although participants are able to choose other modes (e.g. cycling, rowing) if preferred.

#### Remote monitoring

During pre-defined time windows (e.g. 06:00–11:00) participants connect to the remote exercise monitoring system, which permits a remotely-located exercise physiologist to monitor their location, distance, speed, heart rate, respiratory rate, training load and single-lead ECG in real-time; provide real-time feedback and support via participants’ smartphone (including alerts, messages or telephone calls); respond to adverse events if necessary; provide post-exercise feedback; and modify participants’ exercise prescription as required. Participants can be monitored in any environment with an active broadband connection (mobile, Wi-Fi, Bluetooth).

The REMOTE system, comprising a physiological sensing device, smartphone and web apps, and a middleware platform, supports simultaneous monitoring of multiple participants. Participants are loaned a BioHarness 3 (Zephyr Technologies, USA) physiological sensing device to wear during exercise. The BioHarness enables measurement of a comprehensive range of physiological parameters required for monitoring exercise performance. Bluetooth connectivity permits transmission of data to smartphones. The system including the Zephyr device, smartphone (Android) app, and the Odin middleware platform has been tested for reliability and validity (Rawstorn J, Gant N, Warren I, Doughty R, Lever N, Poppe K: Measurement and data transmission validity of a multi-biosensor system for real-time remote exercise monitoring among cardiac patients, forthcoming). The Odin middleware platform is an off-the-shelf solution that provides reliable communication, minimises data usage cost and maximises device battery life [46]. The smartphone app collects and transmits physiological data to a remotely located web server in real-time. The web app displays these data in remote locations, and enables real-time provision of feedback to moderate participants’ exercise behaviour.

#### Support and strategies to facilitate exercise adherence

Messages outlining key behaviour change strategies are sent to participants via the smartphone by the exercise physiologist. Three messages per week are sent for the 12 week intervention period. The program is grounded in self-efficacy theory [47, 48], which is the most examined psychological variable within the cardiac setting. Behaviour change strategies focus on increasing confidence and motivation to exercise, overcoming barriers to being physically active, scheduling exercise into daily life, goal setting, and enhancing social support and networks to be active. In addition, features on the smartphone app allow participants to review their exercise performance and assess progress toward personalised goals.

### Control

The active control group receive an 8-12 week program of supervised exercise, delivered at Auckland or Tauranga CR clinics. Supervised exercise sessions are offered two**-**three times per week by trained exercise scientists/nurses. Participants typically complete a 15-minute warm up, 30–45 minutes of moderate-vigorous intensity aerobic exercise on various exercise modalities (e.g., treadmill, cycle ergometer, rowing machine), and a 5 min cool down. Heart rate, blood pressure, and rating of perceived exertion (RPE) are monitored on a regular basis. Exercise prescription for the control group also adheres to ACSM guidelines for exercise in cardiac patients [43].

### Outcome assessments

All assessments are conducted at the University of Auckland and Tauranga cardiac clinics. Prior to the baseline assessment participants are sent an accelerometer to wear for seven consecutive days. Baseline assessments involve an explanation of study procedures, signed consent and collection of participant-reported secondary outcomes, followed by physical measurements (stature, body mass, body mass index, waist and hip circumference), blood lipid and glucose measurements, and a test of maximal exercise capacity. The baseline assessment concludes with randomisation and assignment of participants to the respective study groups.

The primary outcome is maximal oxygen uptake (V̇O_2_max) assessed at baseline and post-treatment. An individualised exercise testing protocol is used to assess V̇O_2_max via respiratory gas analysis. A graded protocol is initiated at a comfortable walking speed and 0% gradient. The gradient then increases by 1% every 60 seconds until volitional exhaustion, or the presence of indications for test termination [44]. The target exercise time is 8 to 12 minutes. Individualised walking speeds are replicated at the 13 week follow-up assessment. Participants are encouraged to continue the test until the respiratory exchange ratio (RER) exceeds 1.10, indicative of a maximal aerobic performance. The ACSM guidelines for exercise testing cardiac patients are adhered to [44]. Exercise testing is conducted at the University of Auckland Clinics, Tamaki Campus, and the Tauranga cardiac clinic by trained physiologists. A medical physician is available to deal with any emergencies that may arise.

### Secondary outcomes

All secondary outcomes are assessed at baseline, post-treatment and 6 months, except for blood lipid and glucose concentrations, which are assessed at baseline and post-treatment only. Stature, body mass, waist and hip circumference, and blood pressure are measured using standardised procedures. An electronic sphygmomanometer will be used to assess systolic and diastolic pressure after at least 5 minutes of seated rest. Participants’ height will be measured to the nearest 0.1 cm using a stadiometer, and body mass to the nearest 0.1 kilograms using electronic scales. Body mass index is derived from the weight (kg) divided by height (m) squared. Waist circumference is measured using an anthropometric tape measure placed around the participant’s waist at the level of the umbilicus. Hip circumference is measured around the furthest protrusion of the buttocks as seen from a lateral perspective. Waist-hip ratio is calculated according to ISAK protocols [49].

A fingertip blood sample is obtained from participants for analysis of blood lipid and glucose concentration using automated point of care analysers (Cholestech LDX at the Auckland site, CardioChek at the Tauranga site).

Psychological variables including self-efficacy (situational self-confidence), intentions, and motivation to exercise are assessed to determine their potential mediating effect. Task self-efficacy is assessed using a scale adapted from the Self-Efficacy Scale [50]. Participants rate their confidence to perform physical activities for increasing periods of time (i.e., 10, 30, and 60 min) at three intensities (i.e., light, moderate, and vigorous). A key is provided to define these levels of intensity. Mean scores are calculated with higher values indicating greater efficacy to perform physical activity for longer duration and greater intensity.

Participants’ confidence to exercise in the face of obstacles (barrier efficacy) is assessed using the Barriers Efficacy Scale [50]. Participants rate their confidence to overcome eight common reasons preventing people from participating in exercise sessions (e.g., pain, bad weather) on a scale ranging from 0% (no confidence at all) to 100% (completely confident). Mean scores are calculated with higher values indicating greater efficacy to overcome barriers to exercise.

Self-efficacy to follow their exercise prescription is assessed using 9 items. Participants rate their confidence in their ability to follow the prescribed exercise regimen on a scale ranging from 0% (no confidence at all) to 100% (completely confident). Mean scores are calculated with higher values indicating greater efficacy to adhere to prescribed exercise.

Intentions to perform physical activity are assessed using two items [51], which ask participants to rate their level of intention to follow their exercise prescription during the next 3 months (e.g. “I definitely intend to follow my exercise prescription”). The items are scored using a seven point Likert scale ranging from 0 (completely disagree) to 7 (completely agree). A mean score from the two items is used to give an overall measure of intention with higher values indicating greater intention to perform physical activity.

Self-determination to exercise is operationalised using the Locus of Causality for Exercise Scale; a reliable and valid three-item self-report measure of the extent to which participants feel they choose to exercise with no sense of coercion [52]. Participants rate how much they agree or disagree with each statement on a seven-item Likert scale from 1 (Strongly disagree) to 7 (Strongly agree) indicating their motivation to perform exercise. Mean scores are calculated with higher values indicating greater self-determination or a more internal perceived locus of causality.

Leisure time exercise is assessed using the Godin Leisure Time Physical Activity Questionnaire (GLTPAQ) [53]. This simple three-item questionnaire has well-established reliability and validity and has been used in patients undergoing CR (N = 826) [53].

Objective physical activity is assessed using the Actigraph accelerometer (http://www.theActigraph.com), which is sent to participants to wear for 7 consecutive days. The Actigraph is a small piezoelectric accelerometer and has been validated in healthy and cardiac patients [54, 55]. Raw data are processed according to accepted procedures [56].

Adherence to the prescribed regimens is assessed by determining the number of sessions attended compared to the number of sessions prescribed. Participants in both programs are encouraged to participate in supervised exercise three times per week. Attendance data are collected weekly and summed to determine total attendance (the numerator) compared to total number of sessions prescribed (denominator).

Cost information is collected and includes the cost of each program and direct medical costs (including cost of treatment, primary care, secondary care and over-the-counter medications). The EQ5D [57] is used to obtain a single preference index for calculation of Quality Adjusted Life Year (QALY) to assess cost per QALY for comparison with CR programs. Healthcare utilisation is recorded for adverse events including cardiac events, and other events participants deem likely to be related to their participation in the study (including, but not limited to, musculoskeletal injury).

### Statistical analysis

Statistical analyses will be performed using SAS version 9.2 (SAS Institute Inc. Cary NC) and R version 2.11 (R Foundations for Statistical Computing). Baseline characteristics will be summarised using descriptive statistics. Continuous variables will be described as numbers of observed and missing values, mean, standard deviation, median, minimum and maximum. Categorical variables will be described as frequencies and percentages. Treatment evaluation will be performed on the principle of intention to treat (ITT), using data collected from all randomised participants. Analysis of covariance (ANCOVA) regression model will be used to evaluate the main treatment effect on the primary outcome between the two treatment groups, adjusting for its baseline measure, age, ethnicity and other potential confounding factors (if they are statistically significant at 5% level). A similar approach will be used for other continuous secondary outcome measures. Logistic regression model will be considered for the analysis of a binary outcome (e.g. meeting physical activity recommendations).

## Discussion

This novel study evaluates a supervised exercise program delivered via a mobile phone and sensor system compared to a traditional model of supervised exCR. Remote monitoring may offer the same benefits as supervised gym-based programs, but is potentially more accessible to a broader range of patients and is cost-effective.

The protocol, in accordance with the SPIRIT statement, incorporates findings from recent mobile and telehealth systematic reviews, with the aim of building on this empirical evidence. The REMOTE-CR trial includes an objective assessment of maximal cardiorespiratory fitness, which is the gold standard measurement following exCR. It also includes objective assessment of physical activity, which was lacking in previous research [34].

The non-inferiority trial design is most suitable because it is unlikely the REMOTE intervention will result in substantially greater improvement in exercise capacity compared to traditional supervised exercise [58]. The non-inferiority margin (V̇O_2_ 1.25 ml⋅kg^-1^⋅min^-1^) is clinically significant and is associated with lower cardiovascular mortality [59].
